# Taurolidine Acts on Bacterial Virulence Factors and Does Not Induce Resistance in Periodontitis-Associated Bacteria—An In-Vitro Study

**DOI:** 10.3390/antibiotics9040166

**Published:** 2020-04-07

**Authors:** Sabrina Radakovic, Nicola Andreoli, Simon Schmid, Sandor Nietzsche, Jürg Zumbrunn, Anton Sculean, Sigrun Eick

**Affiliations:** 1 Department of Periodontology, School of Dental Medicine, University of Bern, CH-3010 Bern, Switzerland; sabrina.radakovic@medi.ch (S.R.); nicola.andreoli@students.unibe.ch (N.A.); simon.schmid@students.unibe.ch (S.S.); anton.sculean@zmk.unibe.ch (A.S.); 2 Center for Electron Microscopy, Jena University Hospital, D-07743 Jena, Germany; sandor.nietzsche@med.uni-jena.de; 3 Department of Clinical Affairs R& D, Geistlich Pharma Ag, CH-6110 Wolhusen, Switzerland; Juerg.Zumbrunn@geistlich.ch

**Keywords:** taurolidine, mechanisms of action, development of resistance, minocycline, periodontal pathogens

## Abstract

The aims of the present study were: (a) to determine the mechanism of action of taurolidine against bacterial species associated with periodontal disease, and (b) to evaluate the potential development of resistance against taurolidine as compared with minocycline. After visualizing the mode of action of taurolidine by transmission electron micrographs, the interaction with most important virulence factors (lipopolysaccharide (LPS), *Porphyromonas gingivalis* gingipains, *Aggregatibacter actinomycetemcomitans* leukotoxin), was analyzed. Then, 14 clinical isolates from subgingival biofilm samples were transferred on agar plates containing subinhibitory concentrations of taurolidine or minocycline up to 50 passages. Before and after each 10 passages, minimal inhibitory concentrations (MICs) were determined. Increasing MICs were screened for efflux mechanism. Taurolidine inhibited in a concentration-dependent manner the activities of LPS and of the arginine-specific gingipains; however, an effect on *A. actinomycetemcomitans* leukotoxin was not detected. One *P. gingivalis* strain developed a resistance against taurolidine, which was probably linked with efflux *mechanisms*. An increase of MIC values of minocycline occurred in five of the 14 included strains after exposure to subinhibitory concentrations of the antibiotic. The present results indicate that: (a) taurolidine interacts with LPS and gingipains, and (b) development of resistance seems to be a rare event when using taurolidine.

## 1. Introduction

According to the latest reported data about 42% of the non-institutionalized U.S. population has been diagnosed of periodontitis [1]. Periodontitis is a chronic inflammatory disease, which destructs the tooth’s supporting tissues. In the pathogenesis of periodontitis, the host inflammatory response to subgingival bacteria leading to a pathogenic microbiota plays a central role [2]. Among the pathogenic microbiota, *Porphyromonas gingivalis* has been postulated to act as so called “key stone pathogen” [3] with its major virulence factors are cysteine proteases called gingipains [4]. Other bacteria involved in pathogenesis of periodontitis are *Tannerella forsythia* [5] and *Aggregatibacter actinomycetemcomitans*, which synthetizes a leukotoxin [6]. *A. actinomycetemcomitans* strains differ in their ability to produce leukotoxin; e.g., highly leukotoxin-producing strains (JP2-clone) have a deletion in the promotor region [7].

Antimicrobials are frequently used for the treatment of periodontitis. The adjunctive use of chlorhexidine digluconate may additionally improve the clinical outcomes obtained with conventional mechanical debridement [8], while the use of adjunctive systemic antibiotics appears to be beneficial in the treatment of severe cases of periodontitis [9]. However, the long-term clinical benefit following the use of antibiotics is still unclear [10]. The topical use of minocycline microspheres (a tetracycline derivative) in conjunction with nonsurgical periodontal therapy has been shown to result in additional clinical improvements compared with nonsurgical periodontal therapy alone [11].

One potential alternative antimicrobial agent is taurolidine. Taurolidine is a derivative of the amino acid taurine, as an antimicrobial, it has been proven to be safe and effective for prevention of central venous catheter infection [12]. In vitro-studies indicate an antimicrobial activity against oral microorganisms also when those are organized in a biofilm [13,14]. Its potential application in dentistry has been discussed for several years. It has been shown that rinsing with 2% of taurolidine solution depressed growth of dental biofilm by about 50% [15]. In previous “in vitro” experiments we have evaluated some potential antimicrobial effects of taurolidine. We have shown that the minimal inhibitory concentrations (MIC)s of taurolidine were all below 1 mg/mL taurolidine with the exception of *Candida albicans* [13]. One percent of taurolidine inhibited clearly the formation of 12-species biofilms; however, the effect on an established biofilm was as limited as that of minocycline [16]. In an ex-vivo model using subgingival biofilm samples from periodontitis patients, the decrease of bacterial counts in biofilms was 0.87 log10 cfu, corresponding to 86.5% following the application of 3% taurolidine gel after 60 min [17].

The development of resistance against antimicrobials is meanwhile a global problem, with more than half a million deaths annually being attributed to infections caused by antibiotic-resistant micro-organisms [18]. This is dependent on the used antibiotic; i.e., the relationship is strong when quinolones are used and rather weak when beta-lactams were applied [19]. In addition, a considerable number of studies have reported the development of resistance to commonly used antiseptics, and partly, cross-resistance with antibiotics was also found [20]. The increasing prevalence and spread of antimicrobial resistant bacteria are the natural consequence of genetic bacterial evolution. The more frequently an antimicrobial agent is used, the higher is the probability of resistance formation [21].

Antimicrobial agents’ resistance in bacteria can be of different origin, with a distinction between intrinsic and acquired resistance mechanisms. Intrinsic resistance mechanism is a natural property of microorganisms. Acquired resistance mechanism is based on a genetic modification of the bacterium [22] and it can occur as the result of a mutation or the ingestion of foreign resistance genes. Besides the genetic potential of the microorganism, a present selection pressure of an antimicrobial is of importance, e.g., mutations can be induced by acclimating bacteria to increasing concentrations of antimicrobial agents [22]. Gene transfer, such as the acquisition of extra-chromosomal gene elements by transposons or plasmids, can occur within a few hours [23]. Bacterial antimicrobial resistance mechanisms are based on target alteration, impermeability, enzymatic modification or destruction and efflux [24], in case of resistance to biocides (e.g., chlorhexidine) membrane modification or efflux is involved [25].

The aims of this follow-up study were: a) to determine in more detail the mode of action of taurolidine against bacterial species being associated with periodontal disease, and b) to verify a potential development of resistance against taurolidine as compared to that against minocycline. Our data of the present study suggest an inhibition of lipopolysaccharide (LPS) and of *P. gingivalis* gingipains by taurolidine. In comparison with minocycline, the development of resistance to taurolidine was rare in the in-vitro experiments.

## 2. Results

### 2.1. Visualization of Taurolidine Action

**Figure 1:**
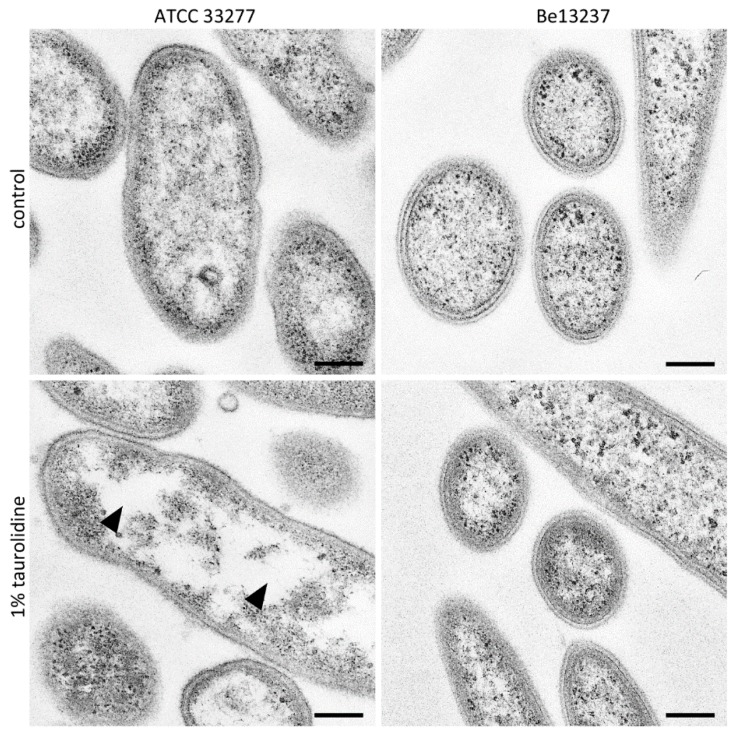
Transmission electron micrographs (TEM) images of *Porphyromonas gingivalis* ATCC 33277 and *Tannerella forsythia* Be13237 after treatment with 1% taurolidine for 2 h, in comparison to its control. *P. gingivalis* shows a decomposition of the cytoplasm leading to unstained areas (arrowheads). For *T. forsythia*, no difference could be found. Scale bars: 200 nm.

Differences of transmission electron micrographs (TEM) of *P. gingivalis* ATCC 33,277 and *T. forsythia* Be13237 without and with 2 h exposure to 1% taurolidine were small (Figure 1). After exposure to taurolidine, a decomposition of the cytoplasm leading to unstained areas within the *P. gingivalis* cells was visible (Figure 1).

### 2.2. Interaction with Most Important Virulence Factors

**Figure 2:**
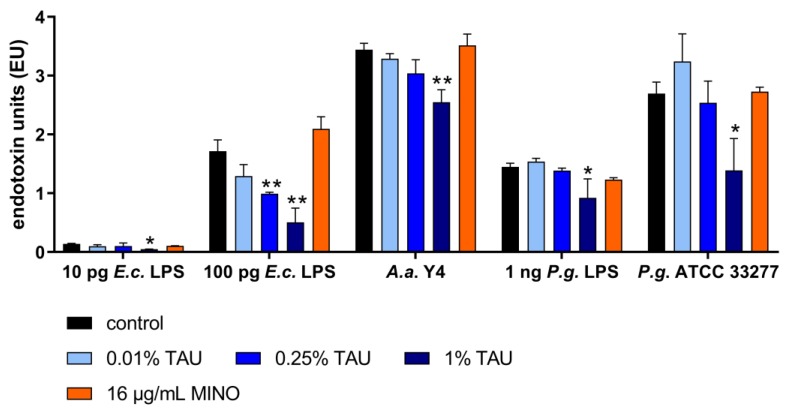
Endotoxin activity (endotoxin units (EU), mean ± SD) measured by Limulus amebocyte lysate assay of *Escherichia coli* (E.c.) LPS, *Porphyromonas gingivalis* (P.g.) LPS as well as *P. gingivalis* ATCC 33,277 and *Aggregatibacter actinomycetemcomitans* Y4, without and with 2 h pre-exposure to 0.01%, 0.25%, 1% taurolidine (TAU) and 16 µg/mL minocycline (MINO).* *p* < 0.05, ** *p* < 0.01 vs. respective control.

Taurolidine inhibited concentration-dependent the endotoxic activity of *Escherichia coli* LPS, *P. gingivalis* LPS, and of *P. gingivalis* ATCC 33,277 and *A. actinomycetemcomitans* Y4. (As *P. gingivalis* LPS has less endotoxin activity [26], a higher concentration was used in comparison with *E. coli* LPS.) Statistically significant differences were always found when comparing 1% taurolidine with control. In contrast, 16 µg/mL of minocycline did not have any effect (Figure 2).

**Figure 3:**
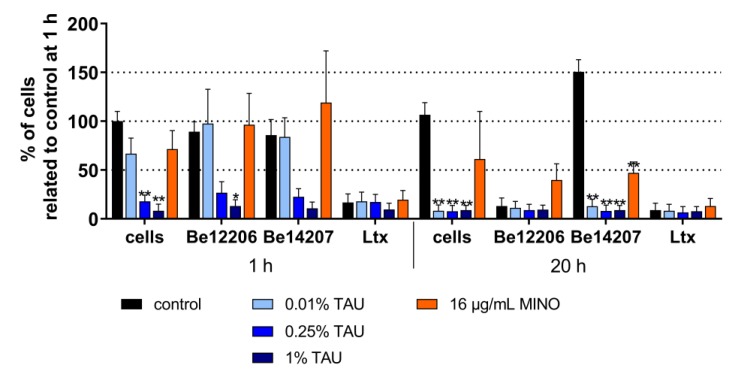
MTT activity (mean ± SD) of monocytic MONO-MAC-6 cells after infection with different *Aggregatibacter actinomycetemcomitans* strains (Be12206, Be14207), after addition of leukotoxin (ltx, 4 µg/mL) and exposure to 0.01%, 0.25%, and 1% taurolidine (TAU) as well as to 16 µg/mL minocycline (MINO).* *p* < 0.05, ** *p* < 0.01 vs. respective control.

Next, the effect of taurolidine on viability of monocytic MONO-MAC-6 cells measured by MTT assay was determined. The results underline the toxicity of purified *A. actinomycetemcomitans* leukotoxin; the viability of the cells was 17%, after 1 h and 8.4% after 20 h related to the non-treated cells. Between the two used bacterial strains, the Be12206 strain belonging to the JP2 clone (well-known for being highly leukotoxic) decreased the viability of the cells to 12.3% after 20 h of incubation. An inhibitory effect of the added antimicrobials (both taurolidine in three concentrations and 16 µg/mL minocycline) on toxicity of leukotoxin to MONO-MAC-6-cells was not seen (Figure 3). In contrast, in this assay an exposure to taurolidine clearly decreased the MTT activity, the effect was visible for 0.25% and 1% of taurolidine already after 1 h and for all three used concentrations after 20 h (each *p* < 0.01).

**Figure 4:**
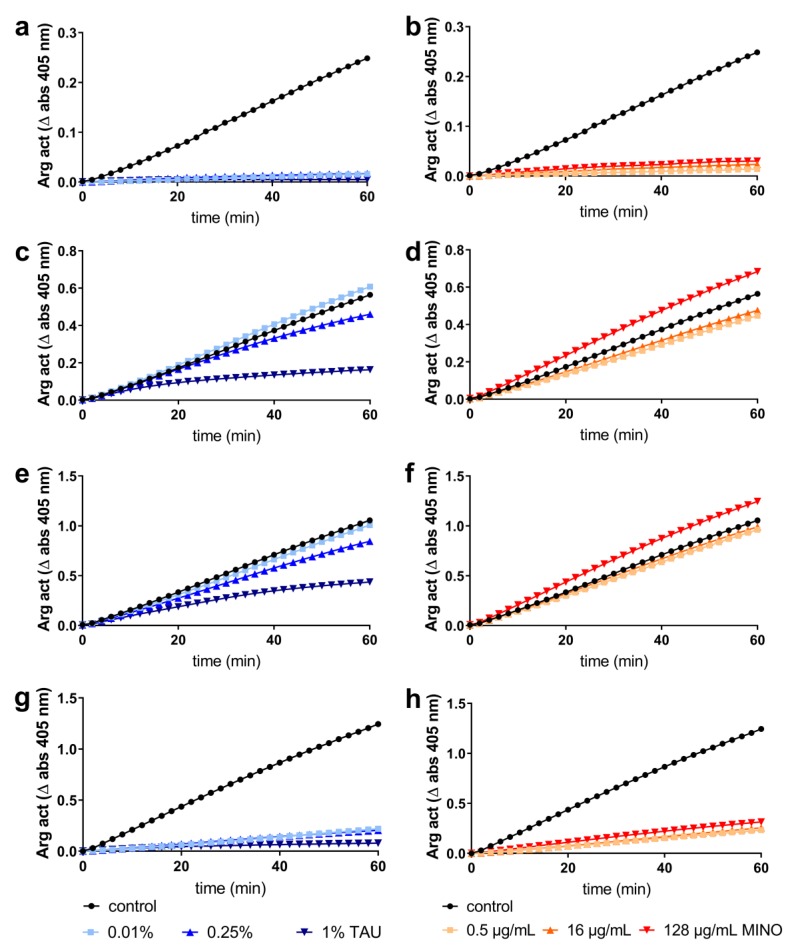
Arginine specific amidolytic activity (Arg act) measured by Δ abs 405 nm after addition of taurolidine (TAU; **a**,**c**,**e**,**g**) and minocycline (MINO; **b**,**d**,**f**,**h**) in different concentrations to 10 nM RgpB (**a**,**b**), *Porphyromonas gingivalis* ATCC 33277 (**c**,**d**), *P. gingivalis* J374-1 (**e**,**f**) and *P. gingivalis* HG66 (**g**,**h**).

Taurolidine blocked the activity of the purified *P. gingivalis* arginine specific cysteine protease (RgpB). Using bacterial strains of *P. gingivalis*, the results were different. Applying 1% taurolidine to *P. gingivalis* ATCC 33277 and J374-1, the measured arginine-specific amidolytic activity clearly decreased, whereas 0.01% did not exert any effect on the enzyme activity. In contrast, when using the HG66 strain, nearly no arginine-specific amidolytic activity was found already after bacterial culture was exposed to 0.01% taurolidine. Results for minocycline were similar for the purified gingipain and the HG66 strain, but there was no inhibition of gingipain activity when using the ATCC 33277 and the J374-1 strains (Figure 4).

### 2.3. Potential Development of Resistance

Analyzing a potential development of resistance, an increase of MIC of taurolidine was found only against one *P. gingivalis* strains after 20 passages. But it is of interest to note that four strains, among them the two included *T. forsythia* strains, became more susceptible to the antimicrobial after certain passages (both *T. forsythia* after 10 passages, one *P. gingivalis* after 20 passages and *Streptococcus constellatus* after 50 passages; Table S1). Resistance development against minocycline was found in five of the 14 included strains after exposure to subinhibitory minocycline (Table S2).

Next, strains exhibiting an altered susceptibility after certain passages were characterized further. The increase of the MIC of taurolidine against one strain (*P. gingivalis* J374-1) might be linked to efflux mechanisms. It seems that this strain contains several efflux pumps. After removing antimicrobial pressure for three passages, MIC values were equal or close to baseline values (Table 1). Efflux mechanisms might play also a role in two *A. actinomycetemcomitans* strains showing an increase in resistance against minocycline. It is obviously not of importance in the other three strains with increased MIC values. Removing antimicrobial pressure for a few passages did not revert the increased MIC values of minocycline (Table 2).

**Figure 5:**
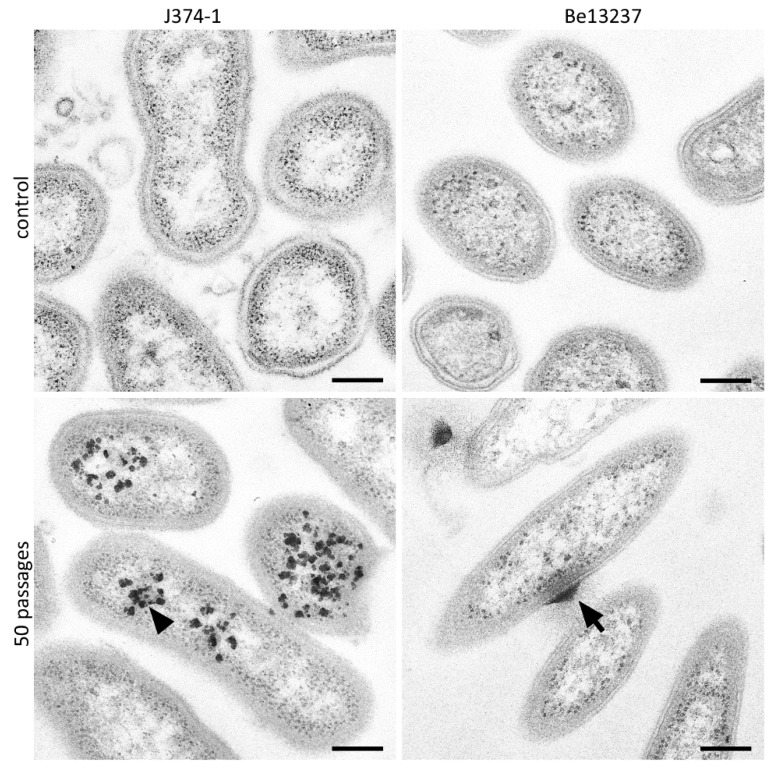
TEM images of *Porphyromonas gingivalis* J374-1ATCC 33,277 and *Tannerella forsythia* Be13237 after 50 passages on agar plates containing subinhibitory concentrations of taurolidine in comparison to its control. *P. gingivalis* J374-1 (a strain becoming more resistant to taurolidine) shows dense particles within cytoplasma (arrowheads). For *T. forsythia* Be13237 (a strain becoming more susceptible) dense structures at the cell wall surrounded by a halo (arrow) were be found. Scale bars: 200 nm.

In the TEM images of *P. gingivalis* J374-1, highly dense particles are visible after exposure to subinhibitory taurolidine. Within the bacterial cells, dense structures are located in the cells. TE micrographs of *T. forsythia* Be13237 show dense structures surrounded by a halo attached to the outer membrane (Figure 5).

## 3. Discussion

In the present study, the mode of action of taurolidine against bacterial species associated with periodontitis and the potential development of resistance were analyzed.

The inhibition of endotoxin activity suggests a potential interaction of taurolidine with the bacterial cell wall. Taurolidine is active against gram-positive and gram-negative bacteria [27]. Taurolidine undergoes several hydrolysis reactions, first a cationic taurolidine is formed [28], which may attack the negatively charged bacterial cell wall. This is a mechanism known for other antimicrobials, e.g., charged chlorhexidine binds to the bacterial cell membranes [29]. For taurolidine, non-isolable carbinolamine is the major bactericidal compound, the antimicrobial activity originates from the methylene iminium ion production [30]. An endotoxin-inactivation has been discussed for many years as taurolidine inhibited concentration-dependent the expression of inflammatory cytokines induced by LPS in peripheral blood mononuclear cells [31]. In contrast, no clear reactivity of taurolidine and its derivatives with peptidoglycan was found [28].

In our study, taurolidine neutralized the endotoxic activity of LPS. This included the LPS derived from *E. coli* and *P. gingivalis* LPS, which showed less endotoxin activity. *P. gingivalis* synthesizes two types of LPS, the O-LPS with O-antigens, and an anionic A-LPS with nonphosphorylated penta-acylated and nonphosphorylated tetra-acylated species, the latter one is less active in stimulating cytotokine production in MONO-MAC-6 cells than total *P. gingivalis* LPS [26].

LPS of *A. actinomycetemcomitans* is involved in the secretion of its leukotoxin, an LPS with altered *O*-antigen polysaccharide leads to an increase of membrane-bound leukotoxin [32]. However, in our study, we have failed to show an inhibition of leukoxin activity by taurolidine. The MTT tetrazolium salt colorimetric assay described by Mosmann (1983) was used to measure viability of MONO-MAC-6 cells; it confirmed the toxicity of leukotoxin and of a highly-leukotoxic *A. actinomycetemcomitans* belonging to the JP2 clone. Viability of the MONO-MAC-6 cells decreased after being in contact with taurolidine. The observed toxicity is in contrast to a report using peripheral blood monocytes where no toxicity was found up to 10% taurolidine [31]. The difference might be explained by the origin of cells. Although MONO-MAC-6 cells are very similar in their expression profile to mature monocytes they originate from a leukemia patient [33].

Except for the *A. actinomycetemcomitans* leukotoxin, we focused on a potential interaction with *P. gingivalis* gingipains. Gingipains are membrane-associated proteases functioning in proteolytic processing of nutrients, adhesion to host cells, evasion of immune response and more attributes [34,35]. The arginine specific amidolytic activity of the purified cysteine protease RgpB and of different strains was clearly inhibited until blocked by taurolidine. Total blocking was found when RgpB and bacterial culture of the HG66 strain were exposed to 1% taurolidine. Thus, it can be concluded that taurolidine inhibits directly gingipain activity. The HG66 strain is the only exception of all *P. gingivalis* strains where gingipains are not glycosylated and bound to outer membranes or outer membrane vesicles but released in a non-glycosylated soluble form into extracellular milieu [4].

In the present study, we compared the potential development of resistance of taurolidine versus minocycline on species being associated with periodontitis. Exposing microbial strains to subinhibitory concentrations of an antimicrobial for certain passages is a widely used method to evaluate the potential of an antimicrobial for development of resistance [36,37]. The results have shown an increase of MIC of taurolidine against only one *P. gingivalis* strain (374-1), whereas a resistance development against minocycline was found in five of the 14 included strains after exposure to subinhibitory concentrations of the respective antimicrobial.

The increase of resistance of taurolidine against one strain (*P. gingivalis* J374-1) might be linked to efflux mechanisms. The efflux seems to be a first line of defense against an antimicrobial by pumping out the antimicrobial and decreasing its level within the bacterial cell [38]. Efflux pumps can be switched on when even low concentrations of an antimicrobial are in the cell, leading to an adaptive resistance, and expression is downregulated when the antimicrobial agent is absent [38]. The process of active efflux is one of the important mechanisms of resistance against antibiotics but also against antiseptics [39]. A *Chryseobacterium indologenes* strain, which was isolated from oral biofilm up-regulated expression of an efflux-pump system in the presence of chlorhexidine digluconate [40]. Higher MIC values of chlorhexidine digluconate against *Pseudomonas aeruginosa* and *Staphylococcus aureus* strains were decreased by adding efflux pump inhibitors [41]. Efflux pumps belong to five families, regarding antimicrobial resistance the ABC pumps in gram-positive bacteria and the RND pumps in gram-negative bacteria are most important [42]. All used inhibitors on efflux pumps increased the sensitivity of the *P. gingivalis* J374-1 strain to taurolidine. Among the used inhibitors, 1-(1-naphthylmethyl)piperazine (NMP) and carbonyl cyanide 3-chlorophenylhydrazone (CCCP) are known to inhibit RND pumps, whereas reserpine and verapamil act on non-RND pumps [43,44]. This leads to the assumption that both RND and non RND-pumps were involved in the adaptive resistance. After three passages of *P. gingivalis* J374-1 on media free of antimicrobials, the MIC of taurolidine, the MICs, became lower than at baseline, which suggests a certain activity of the pumps already before antimicrobial’s exposure.

Interestingly, a few strains became more susceptible to taurolidine when being exposed to subinhibitory concentrations of the antimicrobial. However, this phenomenon is difficult to explain. TEM images depicted areas of halos closed to cell bacterial cell wall leading to the assumption of an enrichment of the antimicrobial there. This might be supported by the fact that the higher susceptibility got lost after a few passages of the strain on agar plates without antimicrobials.

A resistance development against minocycline was found in five of the 14 included strains after exposure to subinhibitory minocycline. As doxycycline, minocycline belongs to the second generation of tetracylines, an antibiotic class, which target is the small subunit of bacterial ribosome where t-RNA binds to the respective nucleic acids of mRNA [45]. Acquired resistance is linked with efflux pumps, but also bacterial proteins removing tetracylines from its binding site, enzymes degrading tetracyclines or mutations within 16S rRNA preventing binding of tetracyclines to ribosomes exist [45]. Resistance to tetracyclines among oral strains is reported differently. Recently about 30% of *Streptococcus intermedius* and *S. constellatus* strains and 47% of the *A. actinomycetemcomitans* were reported to be resistant against doxycycline [46,47]. In contrast, less than 10% of *A. actinomycetemcomitans* strains isolated from aggressive periodontitis patients in Great Britain showed a resistance against tetracyclines [48] and MIC_90_ values were generally low for *P. gingivalis* and for *F. nucleatum* [49]. In two (both *A. actinomycetemcomitans* strains) out of the five strains with higher MIC values to minocycline, the efflux mechanism seem to play a role; in one strain this might be related mainly to RND pumps, in the other strain only to non-RND pumps. In the other three strains (one *T. forsythia*, one *F. nucleatum* and one oral streptococcus), other mechanisms, which have not been investigated in detail, may account for the increased MIC values. However, it is of interest to note that removing antimicrobial pressure for a few passages did not revert the increased MIC values. In a previous study, most oral bacteria survived transitory 2–8-fold MIC values of minocycline but finally, the MIC values were not different from those at baseline (at the highest one step) [50].

## 4. Materials and Methods

### 4.1. Antimicrobials

In all experiments, taurolidine 2% solution (Geistlich Pharma AG, Wolhusen, Switzerland) was used and diluted until to the necessary dilution. As positive control minocycline (Sigma-Aldrich, Buchs, Switzerland) and as negative control dH_2_O were used.

### 4.2. Microorganisms

Several oral bacterial strains (laboratory strains and clinical isolates; mainly *P. gingivalis* and *A. actinomycetemcomitans*) were included in the experiments (Table S3). The clinical isolates (4 *P. gingivalis*, 3 *A. actinomycetemcomitans*, 2 *T. forsythia*, 2 *Fusobacterium nucleatum*, 4 oral streptococci) were obtained from subgingival biofilm samples. Samples were only obtained from individuals who did not receive antibiotic treatment 2 months prior to the date of collecting. Culturing subgingival biofilm and isolation of respective bacterial strains was approved by the Ethical Committee of the Canton Bern (KEK 096/15). Without removing of supragingival biofilm, sterile paper point (ISO 50) were inserted into the periodontal pocket for 30 s. Then, the pooled paper points were placed into 1 mL of reduced transport fluid. After culturing on tryptic-soy-agar-plates with 5% of sheep blood (Oxoid, Basingstoke, GB) under anaerobic conditions for 8 d, colonies typical for the respective species were isolated and subcultivated. Identity was confirmed by PCR using species-specific primers [51].

Bacterial strains were kept frozen at −80 °C. About one week before experiments, they were subcultured and passaged 2–3 times on tryptic-soy-agar plates with 5% of sheep blood.

### 4.3. Cells

Monocytic cells of human origin (MONO-MAC-6; DSMZ no. ACC 124) were maintained in RPMI 1640 medium containing 10% fetale bovine serum (FBS). For experiments, cells were used in M199 media without FBS (Invitrogen; Carlsbad, CA, USA).

### 4.4. TEM Images after Exposure to 1% Taurolidine

Two bacterial strains (*P. gingivalis* ATCC 33277, *T. forsythia* B13237) were included. They were suspended to a density of about McFarland 4 in nutrient broth (Wilkins–Chalgren broth (Oxoid) supplemented with 5 mg/l β-NAD and 10 mg/l NAM (both Sigma-Aldrich, Buchs, Switzerland)) without and with 1% of taurolidine. After an incubation for 2 h in an anaerobic atmosphere at 37 °C, these bacteria were prepared for transmission electron microscopy (TEM).

For TEM, bacteria suspensions were fixed with 0.5% formaldehyde and 2% glutaraldehyde in 0.1 M cacodylate buffer (pH 7.4) for 30 min. After washing 3-fold with 0.1 M cacodylate buffer 10 min each and post-fixating with 1% osmiumtetroxide for 1 h, dehydration in ascending ethanol series (30%, 50%, 70%, 80%, 90%, 100%, 10 min each) was performed. Moreover, after, the 50% stage post-staining with 2% uranylacetate in 50% ethanol for 2 h in the dark was conducted. Afterwards, the samples were embedded in epoxy resin (Araldite) and sectioned using a Leica Ultracut E (Leica, Wetzlar, Germany). Ultrathin sections (60 nm thickness) were mounted on filmed Cu grids (Formvar/carbon film, Quantifoil Micro Tools GmbH, Jena, Germany), post-stained with lead citrate for 5 min, and studied in a transmission electron microscope (EM 900, Zeiss, Oberkochen, Germany) at 80 kV and magnifications of 3,000× to 20,000×. All preparation steps were performed at room temperature.

### 4.5. Interaction with Bacterial Virulence Factors

To determine a potential effect of taurolidine on lipopolysaccharide (LPS), 10 pg and 100 pg LPS originated from *Escherichia coli* (Sigma-Aldrich) and 10 ng LPS from *P. gingivalis* (InvivoGen, Toulouse, France) were used. Taurolidine in the final concentrations of 0.01% *w*/*v*, 0.25% *w*/*v* and 1% *w*/*v* and as control 16 µg/mL minocycline were added to the LPS for 2 h at 37 °C. Thereafter, endotoxin activity was measured by Limulus amebocyte lysate assay (LAL) QCL-1000^TM^ test (Lonza, Basel, Switzerland) according to the manufacturer’s recommendation. Moreover, the two bacterial strains *P. gingivalis* ATCC 33,277 and *A. actinomycetemcomitans* Y4 (about 10^8^/mL) were exposed to the same concentrations of antimicrobials for 2 h before measuring endotoxin activity.

The leukotoxin was purified as described by Kachlany et al. [52], by adding a final centrifugation using 10 kDa centrifugal filter to remove proteins of lower weights. Leukotoxin in a concentration of 4 µg/mL and two *A. actinomycetemcomitans* strains (10^8^/mL in M199 media) were exposed to three concentrations of taurolidine (0.01% *w*/*v*, 0.5% *w*/*v*, 1.5% *w*/*v*) and to 16 mg/mL minocycline for 2 h before monocytic cells (10^6^/mL in M199 media) were added in a ratio 1:1. *A. actinomycetemcomitans* leuktoxin is well known to affect viability of leukocytes, such as monocytes [6].) Viability of MONO-MAC-6 cells was determined after 1 h and 20 h of incubation at 37 °C with 5% of CO_2_ by using ((3-(4,5-dimethylthiazol-2-yl)-2,5-diphenyltetrazolium bromide) tetrazolium) MTT assay according to Mosmann [53], which quantifies the amount of MTT reduced by the cells to its formazan derivative.

Experiments on LPS and leukotoxin activity were made in independent triplicates. One-way ANOVA with post-hoc Bonferroni compared different antimicrobials with controls each. Further, t-test was used to find differences in viability of MONO-MAC-6 cells after exposing *A. actinomycetemcomitans* strains and leukotoxin with non-stimulate cells (only controls). A difference with a *p*-value of < 0.05 was considered as being statistically significant each.

A direct inhibitory effect on gingipain activity was assessed by adding three concentrations of taurolidine (0.01% *w*/*v*, 0.25% *w*/*v*, 1% *w*/*v*) and of minocycline (0.5 µg/mL, 16 µg/mL, 128 µg/mL) for 2 h to 10 nM activated RgpB (being an arginine-specific gingipain; the RgpB was kindly provided by Jan Potempa, Jagiellonian University Krakow, Poland). Thereafter, *P. gingivalis* strains (10^8^/mL Wilkins–Chalgren broth) were exposed to the same concentrations of the antimicrobials for 2 h (anaerobic atmosphere, 37 °C) before measuring arginine specific amidolytic activity in the suspensions by adding the chromogenic *N*-α-benzoyl-DL-arginine-*p*-nitroanilide (BA*p*NA) substrate (Sigma-Aldrich), USA), with a final concentration of 2 mM in the assay buffer (0.2M Tris-HCl, 0,1M NaCl, 5 mM CaCl_2_, pH 7.6, supplemented with 10 nM cysteine hydrochloride solution in a ratio 1:1. The absorbance was read at 405 nm (37 °C) at 30 s intervals for 1 h by using a spectrophotometer (BioTek EL808, BioTek, Luzern, Switzerland).

### 4.6. Potential Development of Resistance

Initially, all clinical isolates were included in this experiment. However, the *A. actinomycetemcomitans* Be12206 strain did not survive the passages. Therefore, only results from 14 strains were available. The method was adapted to the procedures made before [37,54]. For that, strains were transferred on Wilkins–Chalgren agar plates (Oxoid) containing 1/4–1/8 of the MICs of taurolidine or minocycline up to 50 passages of the respective strain (about one passage each five days). Before and after each 10 passages, MICs were determined again by agar dilution technique (Wilkins–Chalgren agar plates plus respective concentration of the antimicrobial). If there was an increase or decrease of MICs after passaging, the concentration of the antimicrobial was adapted. Further strains with altered MICs were passaged 3 times on media free of antimicrobials to see the stability of the altered MIC. Strains showing changed MIC before and after 50 passages with subinhibitory concentrations of taurolidine were prepared for TEM and SEM, as described above.

Strains with increased MICs were screened for potential efflux resistance mechanisms. The efflux inhibitors 50 µg/mL NMP, 10 µg/mL CCCP, 100 µg/mL verapamil, and 20 µg/mL reserpine (all inhibitors Sigma-Aldrich) were added to the nutrient media (Wilkins–Chalgren broth) together with antimicrobials. The MICs were determined after 18 h of incubation.

## 5. Conclusions

Taurolidine interacts with LPS of periodontopathogens and inhibits the activity of gingipains. Since the development of resistance against taurolidine occurs very rarely when compared with that induced by minocycline, further studies are warranted to evaluate its potential use in the treatment of periodontal and peri-implant infections.

## Figures and Tables

**Table 1:** MIC of taurolidine (% *w*/*v*) of strains with changed MIC values after 50 passages on subinhibitory concentrations of taurolidine. Presented are MIC values before and after 50 passages, after addition of efflux inhibitors reserpine, NMP, CCCP, verapamil (only strain showing increase of MIC) as well as after additional three passages on agar plates free of antimicrobials.

Strain	Baseline	After 50 Pass.	With 20 µg/mL Reserpine	With 50 µg/mL NMP	With 10 µg/mL CCCP	With 100 µg/mL Verapamil	After 3 pass. w/o Taurolidine
*P. gingivalis* J374-1	0.025	0.1	0.013	0.013	0.006	0.006	0.025
*S. constellatus* BeTa7-1	0.1	0.025	n.d.	n.d.	n.d.	n.d.	0.05
*T. forsythia* Be13237	0.025	≤0.003	n.d.	n.d.	n.d.	n.d.	0.025
*T. forsythia* Be13216	0.025	≤0.003	n.d.	n.d.	n.d.	n.d.	0.013

pass. = passages; n.d. = not done.

**Table 2:** MIC of minocycline (µg/mL) of strains with changed MIC values after 50 passages on subinhibitory concentrations of minocycline. Presented are MIC values before and after 50 passages, after addition of efflux inhibitors reserpine, NMP, CCCP, verapamil, as well as after additional three passages on agar plates free of antimicrobials

Strain	Baseline	After 50 pass.	With 20 µg/mL reserpine	With 50 µg/mL NMP	With 10 µg/mL CCCP	With 100 µg/mL verapamil	After 3 pass. w/o MINO
*Streptococcus oralis* JM933	1	4	4	4	4	4	4
*A. actinom.* OMZ 444	1	32	8	≤0.5	≤0.5	8	32
*A. actinom.* Be14207	8	32	8	16	32	4	32
*Fusobacterium nucleatum* BeTa9-1	1	4	4	4	4	4	4
*T. forsythia* Be13216	0.5	2	2	2	2	2	2

pass. = passages; A. actinom. = A. actinomycetemcomitans.

## Supplementary Materials

The following are available online at https://www.mdpi.com/2079-6382/9/4/166/s1, Table S1: Minimal inhibitory concentration (MIC) of taurolidine (% *w*/*v*) determined at baseline and after certain passages on agar plates containing subinhibitory concentrations of the compound; Table S2: Minimal inhibitory concentration (MIC) of minocycline (µg/mL) at baseline and after certain passages on agar plates containing subinhibitory concentrations of the compound; Table S3: Strains included in the experiments.
