# Supplementary material for: Taurolidine Acts on Bacterial Virulence Factors and Does Not Induce Resistance in Periodontitis-Associated Bacteria—An In-Vitro Study
Source: Antibiotics (Basel). 2020 Apr 7;9(4):166. doi: 10.3390/antibiotics9040166 (PMC7235838; doi:10.3390/antibiotics9040166)
Supplement: Supplementary file 1 [file antibiotics-09-00166-s001.pdf]

Table S1
Minimal inhibitory concentration (MIC) of taurolidine (% w/v) determined at baseline and after certain passages on agar plates containing subinhibitory concentrations of the compound

	Base-line	After 10 passages	After 20 passages	After 30 passages	After 40 passages	After 50 passages
***Porphyromonas gingivalis* BeOR14**	0.025	0.025	0.025	0.025	0.025	0.025
***P. gingivalis* BeOR6**	0.025	0.05	0.05	0.05	0.05	0.05
***P. gingivalis* BeOR15**	0.025	0.025	0.025	0.025	0.025	0.025
***P. gingivalis* J374-1**	0.025	0.025	0.1	0.1	0.1	0.1
***Streptococcus constellatus* BeTa7-1**	0.1	0.1	0.1	0.1	0.1	0.025
***S. mitis* BeTa7-2**	0.05	0.05	0.05	0.05	0.05	0.05
***S. gordonii* BeTa9-2**	0.1	0.1	0.1	0.1	0.1	0.05
***S. oralis* JM933**	0.1	0.1	0.1	0.1	0.1	0.1
***Aggregatibacter actinomycetemcomitans* OMZ 444**	0.1	0.1	0.1	0.1	0.1	0.05
***A. actinom.* Be14207**	0.05	0.05	0.05	0.05	0.05	0.05
***Fusobacterium nucleatum* BeOR1**	0.05	0.05	0.05	0.05	0.05	0.05
***F. nucleatum* BeTa9-1**	0.05	0.1	0.1	0.1	0.1	0.1
***Tannerella forsythia* Be13237**	0.025	0.003	0.003	0.003	0.003	0.003
***T. forsythia* Be13216**	0.025	0.003	0.003	0.003	0.003	0.003

Table S2
Minimal inhibitory concentration (MIC) of minocycline (µg/ml) at baseline and after certain passages on agar plates containing subinhibitory concentrations of the compound

	Base-line	After 10 passages	After 20 passages	After 30 passages	After 40 passages	After 50 passages
***P. gingivalis* BeOR14**	1	1	1	1	1	0.5
***P. gingivalis* BeOR6**	1	1	1	1	1	1
***P. gingivalis* BeOR15**	1	1	1	1	1	1
***P. gingivalis* J374-1**	1	1	1	1	1	1
***S. constellatus* BeTa7-1**	8	8	8	8	8	16
***S. mitis* BeTa7-2**	8	8	16	16	16	16
***S. gordonii* BeTa9-2**	16	16	16	16	16	16
***S. oralis* JM933**	1	1	4	4	4	4
***A. actinom* OMZ 444**	1	2	16	32	32	32
***A. actinom.* Be14207**	8	16	16	32	32	32
***F. nucleatum* BeOR1**	1	1	1	1	1	1
***F. nucleatum* BeTa9-1**	1	1	1	8	8	4
***T. forsythia* Be13237**	0.5	0.25	0.25	0.25	0.25	0.25
***T. forsythia* Be13216**	0.5	2	2	2	2	2

Table S3
Strains included in the experiments

Species	Strain	Origin	Gram property
P. gingivalis	ATCC 33277	Laboratory	Negative
P. gingivalis	HG66	Laboratory	Negative
P. gingivalis	J374-1	Clinical	Negative
P. gingivalis	BeOR6	Clinical	Negative
P. gingivalis	BeOR14	Clinical	Negative
P. gingivalis	BeOR15	Clinical	Negative
Aggregatibacter actinomycetemcomitans	Y4	Laboratory	Negative
A. actinomycetemcomitans	Be12206	Clinical	Negative
A. actinomycetemcomitans	OMZ 444	Clinical	Negative
A. actinomycetemcomitans	Be14207	Clinical	Negative
Fusobacterium nucleatum	BeOR1	Clinical	Negative
F. nucleatum	BeTa9-1	Clinical	Negative
Tannerella forsythia	Be13237	Clinical	Negative
T. forsythia	Be13216	Clinical	Negative
Parvimonas micra	ATCC 33270	Laboratory	Positive
Streptococcus oralis	BeJM933	Clinical	Positive
S. constellatus	BeTa7-1	Clinical	Positive
S. mitis	BeTa7-2	Clinical	Positive
S. gordonii	BeTa9-2	Clinical	Positive
